# Decreasing time to antiretroviral therapy initiation after HIV diagnosis in a clinic‐based observational cohort study in four African countries

**DOI:** 10.1002/jia2.25446

**Published:** 2020-02-17

**Authors:** Allahna L Esber, Peter Coakley, Julie A Ake, Emmanuel Bahemana, Yakubu Adamu, Francis Kiweewa, Jonah Maswai, John Owuoth, Merlin L Robb, Christina S Polyak, Trevor A Crowell, for the AFRICOS Study Group, A Parikh, J Hern, O Falodun, K Song, M Milazzo, N Dear, L Francisco, S Mankiewicz, S Schech, A Golway, T Mebrahtu, E Lee, K Bohince, T Hamm, K Lombardi, M Imbach, L Eller, S Peel, J Malia, A Kroidl, I Kroidl, C Geldmacher, C Kafeero, A Nambuya, J Tegamanyi, H Birungi, O Mugagga, G Nassali, P Wangiri, M Nantabo, P Nambulondo, B Atwijuka, A Asiimwe, CT Nabanoba, M Semwogerere, R Mwesigwa, S Jjuuko, R Namagembe, E Bagyendagye, A Tindikahwa, I Rwomushana, F Ssentongo, H Kibuuka, M Millard, J Kapkiai, S Wangare, R Mangesoi, P Chepkwony, L Bor, E Maera, A Kasembeli, J Rotich, C Kipkoech, W Chepkemoi, A Rono, Zeddy Kesi, Janet Ngeno, Edwin Langat, Keneddy Labosso, Ken Langat, Robert Kirui, L Rotich, M Mabwai, E Chelangat, J Agutu, C Tonui, E Changwony, M Bii, E Chumba, J Korir, J Sugut, D Gitonga, R Ngetich, S Kiprotich, W Rehema, C Ogari, I Ouma, O Adimo, S Ogai, C Okwaro, E Maranga, J Ochola, K Obambo, V Sing'oei, L Otieno, O Nyapiedho, N Sande, E Odemba, F Wanjiru, S Khamadi, E Chiweka, A Lwilla, D Mkondoo, N Somi, P Kiliba, M Nwando, G Mwaisanga, J Muhumuza, N Mkingule, O Mwasulama, A Sanagare, P Kishimbo, G David, F Mbwayu, J Mwamwaja, J Likiliwike, J Muhumuza, R Mcharo, N Mkingule, O Mwasulama, B Mtafya, C Lueer, A Kisinda, T Mbena, H Mfumbulwa, L Mwandumbya, P Edwin, W Olomi, Y Adamu, A Akintunde, AB Tiamiyu, K Afoke, S Mohammed, NE Harrison, UC Agbaim, OA Adegbite, Z Parker, GA Adelakun, FO Oni, RO Ndbuisi, J Elemere, N Azuakola, TT Williams, M Ayogu, O Enas, O Enameguono, AF Odo, IC Ukaegbu, O Ugwuezumba, SO Odeyemi, NC Okeke, L Umeji, A Rose, H Daniel, H Nwando, EI Nicholas, T Iyanda, C Okolo, VY Mene, B Dogonyaro, O Olabulo, O Akinseli, F Onukun, G Knopp

**Affiliations:** ^1^ U.S. Military HIV Research Program Walter Reed Army Institute of Research Silver Spring MD USA; ^2^ Henry M. Jackson Foundation for the Advancement of Military Medicine Bethesda MD USA; ^3^ Henry Jackson Foundation MRI Mbeya Tanzania; ^4^ U.S. Army Medical Research Directorate – Africa Nairobi Kenya; ^5^ Henry Jackson Foundation MRI Abuja Nigeria; ^6^ Makerere University Walter Reed Project Kampala Uganda; ^7^ Kenya Medical Research Institute/U.S. Army Medical Research Directorate Nairobi Kenya; ^8^ Henry Jackson Foundation MRI Kericho Kenya; ^9^ Henry Jackson Foundation MRI Kisumu Kenya

**Keywords:** antiretroviral therapy, highly active, HIV‐1, treatment initiation, Africa, time‐to‐treatment, CD4 lymphocyte count

## Abstract

**Introduction:**

World Health Organization (WHO) guidelines have shifted over time to recommend earlier initiation of antiretroviral therapy (ART) and now encourage ART initiation on the day of HIV diagnosis, if possible. However, barriers to ART access may delay initiation in resource‐limited settings. We characterized temporal trends and other factors influencing the interval between HIV diagnosis and ART initiation among participants enrolled in a clinic‐based cohort across four African countries.

**Methods:**

The African Cohort Study enrols adults engaged in care at 12 sites in Uganda, Kenya, Tanzania and Nigeria. Participants provide a medical history, complete a physical examination and undergo laboratory assessments every six months. Participants with recorded dates of HIV diagnosis were categorized by WHO guideline era (<2006, 2006 to 2009, 2010 to 2012, 2013 to 2015, ≥2016) at the time of diagnosis. Cox proportional hazard modelling was used to estimate hazard ratios (HRs) and 95% confidence intervals (95% CI) for time to ART initiation.

**Results and discussion:**

From January 2013 to September 2019, a total of 2888 adults living with HIV enrolled with known diagnosis dates. Median time to ART initiation decreased from 22.0 months (interquartile range (IQR) 4.0 to 77.3) among participants diagnosed prior to 2006 to 0.5 months (IQR 0.2 to 1.8) among those diagnosed in 2016 and later. Comparing those same periods, CD4 nadir increased from a median of 166 cells/mm^3^ (IQR: 81 to 286) to 298 cells/mm^3^ (IQR: 151 to 501). In the final adjusted model, participants diagnosed in each subsequent WHO guideline era had increased rates of ART initiation compared to those diagnosed before 2006. CD4 nadir ≥500 cells/mm^3^ was independently associated with a lower rate of ART initiation as compared to CD4 nadir <200 cells/mm^3^ (HR: 0.32; 95% CI: 0.28 to 0.37). Age >50 years at diagnosis was independently associated with shorter time to ART initiation as compared to 18 to 29 years (HR: 1.38; 95% CI: 1.19 to 1.61).

**Conclusions:**

Consistent with changing guidelines, the interval between diagnosis and ART initiation has decreased over time. Still, many adults living with HIV initiated treatment with low CD4, highlighting the need to diagnose HIV earlier while improving access to immediate ART after diagnosis.

## 1 Introduction

The introduction of antiretroviral therapy (ART) proved a turning point in the fight against HIV, leading to dramatic decreases in HIV‐related morbidity and mortality 1, 2. However, the first ART regimens carried large pill burdens and were poorly tolerated due to toxicities such as lipodystrophy and severe anaemia 3. To avoid complications of therapy and reduce costs, international guidelines initially recommended delaying ART initiation until CD4 was <200 cells/mm^3^. However, evidence of the benefit of earlier ART initiation has gradually accumulated over time. For example, studies have shown only partial restoration of CD4 when ART is initiated at a low CD4 count, whereas earlier initiation effectively prevents CD4 decline in the first place 4, 5. Early initiation of suppressive ART also prevents onward HIV transmission to uninfected partners 6, reduces AIDS‐defining and non‐AIDS‐defining clinical events 7, and decreases mortality 8. Concurrent with this evolving knowledge of ART benefits, regimens have become more readily available, easier to take and better tolerated, with multiple single‐tablet once‐daily regimens now available that have few adverse effects 3, 9.

Accordingly, World Health Organization (WHO) guidelines have shifted over the years to recommend earlier initiation of ART. Prior to 2006, WHO recommended waiting to initiate ART until CD4 declined to <200 cells/mm^3^ 10. In 2006, this was adapted to also consider initiation with CD4 201 to 350 cells/mm^3^ depending on clinical stage. The ART guidelines for adults were changed again in 2010 to recommend ART initiation at CD4 <350 cells/mm^3^ irrespective of clinical stage, and in 2013 this threshold was increased to ≤500 cells/mm^3^ 10. Beginning in 2016, WHO recommended rapid initiation of ART at the time of HIV diagnosis regardless of CD4 count 11. However, barriers to ART access may delay initiation in resource‐limited settings such as sub‐Saharan Africa. Understanding the extent to which these guidelines have impacted HIV care in real world settings is crucial to achieving an AIDS‐free generation. The objective of these analyses was to characterize temporal trends and other factors influencing the interval between HIV diagnosis and ART initiation in four African countries.

## 2 Methods

### 2.1 Study population

These analyses used data from the African Cohort Study (AFRICOS), a longitudinal cohort enrolling adults engaged in care at 12 sites in Uganda, Kenya, Tanzania and Nigeria supported by the President's Emergency Plan for AIDS Relief (PEPFAR). AFRICOS participants provided a medical history, completed a physical examination and underwent laboratory assessments every six months as described previously 12. HIV diagnosis dates, ART start dates and CD4 nadir were obtained from medical record review. For participants with only a diagnosis or ART initiation year, 1 July was used as the month/date. The 1^st^ was used if only the ART initiation day was missing. All participants with a known HIV diagnosis year were eligible for these analyses.

### 2.2 Analysis

All data were recorded on paper case report forms and double entered into the ClinPlus platform (DZS Software Solutions, Bound Brock, NJ). Analyses were performed in SAS 9.3 (SAS, Cary, NC) and Stata 14.0 (StataCorp, College Station, TX).

Participants were categorized by WHO guideline era (<2006, 2006 to 2009, 2010 to 2012, 2013 to 2015, ≥2016) at the time of HIV diagnosis. These eras were based on the release dates of major guideline updates. Cox proportional hazard modelling was used to estimate unadjusted and adjusted hazard ratios (HRs) for factors potentially associated with time to ART initiation, including WHO guideline era, WHO stage at diagnosis, CD4 nadir (<200, 200 to 349, 350 to 499, ≥500 cells/mm^3^), country (Uganda, Kenya, Tanzania, and Nigeria), age at diagnosis, gender and education. Backwards selection was used to select the final adjusted model. While our models did not meet the assumptions of proportional hazards using Schoenfeld residuals, visual inspection of the plots suggested no major violations. Participants were censored at the last available study visit before loss to follow‐up (no contact for 360 days after missed visit), death or data cut‐off of 1 September 2019. To ensure the robustness of our data, two sensitivity analyses were performed. First, the model was restricted to only participants with complete dates (day, month and year) of HIV diagnosis and ART initiation. We also reran the models using calendar year of HIV diagnosis rather than WHO guideline era as a categorical independent variable. Due to small sizes, for this model we combined all years prior to 2006 (n = 237) and after 2016 (n = 167).

### 2.3 Ethical considerations

The study was approved by institutional review boards of the Walter Reed Army Institute of Research, Makerere University School of Public Health, Kenya Medical Research Institute, Tanzania National Institute of Medical Research, and Nigerian Ministry of Defence. All participants provided written informed consent prior to enrolment.

## 3 Results and discussion

From 23 January 2013 to 1 September 2019, a total of 2930 people living with HIV (PLHIV) were enrolled in AFRICOS. Forty‐two did not have a known HIV diagnosis date while 60 only had a diagnosis year and 117 had month and year. Of the 2888 participants with a known HIV diagnosis date, 767 (26.6% were aged 18 to 29 years and 1688 (58.4%) were female (Table 1). ART was initiated in 2747 (95.1%) participants.

**Table 1:** Participant characteristics by WHO guideline era at HIV diagnosis

	All participants (N = 2888)	WHO guideline era at HIV diagnosis				
		<2006 (N = 237)	2006 to 2009 (N = 722)	2010 to 2012 (N = 535)	2013 to 2015 (N = 964)	2016 (N = 430)
Age at diagnosis, years						
18 to 29	767 (26.6%)	90 (38.0%)	184 (25.5%)	149 (27.9%)	225 (23.3%)	119 (27.7%)
30 to 39	1121 (38.8%)	91 (38.4%)	290 (40.2%)	196 (36.6%)	377 (39.1%)	167 (38.8%)
40 to 49	732 (25.3%)	44 (18.6%)	190 (26.3%)	139 (26.0%)	260 (27.0%)	99 (23.0%)
50+	268 (9.3%)	12 (5.1%)	58 (8.0%)	51 (9.5%)	102 (10.6%)	45 (10.5%)
Gender						
Male	1200 (41.6%)	92 (38.8%)	289 (40.0%)	238 (44.5%)	406 (42.1%)	175 (40.7%)
Female	1688 (58.4%)	145 (61.2%)	433 (60.0%)	297 (55.5%)	558 (57.9%)	255 (59.3%)
Country						
Uganda	528 (18.3%)	44 (18.6%)	126 (17.5%)	62 (11.6%)	196 (20.3%)	100 (23.3%)
Kenya	1498 (51.9%)	119 (50.2%)	410 (56.8%)	355 (66.4%)	471 (48.9%)	143 (33.3%)
Tanzania	563 (19.5%)	55 (23.2%)	128 (17.7%)	57 (10.7%)	159 (16.5%)	164 (38.1%)
Nigeria	299 (10.4%)	19 (8.0%)	58 (8.0%)	61 (11.4%)	138 (14.3%)	23 (5.3%)
Education						
None or some primary	947 (32.8%)	63 (26.6%)	232 (32.1%)	173 (32.3%)	356 (36.9%)	123 (28.6%)
Primary or some secondary	1152 (39.9%)	76 (32.1%)	303 (42.0%)	209 (39.1%)	362 (37.6%)	202 (47.0%)
Secondary and above	786 (27.2%)	98 (41.4%)	186 (25.8%)	152 (28.4%)	245 (25.4%)	105 (24.4%)
Missing	3 (0.1%)	0 (0.0%)	1 (0.1%)	1 (0.2%)	1 (0.1%)	0 (0.0%)
CD4 nadir, cells/mm^3^						
<200	1288 (44.6%)	141 (59.5%)	398 (55.1%)	250 (46.7%)	363 (37.7%)	136 (31.6%)
200 to 349	774 (26.8%)	51 (21.5%)	221 (30.6%)	157 (29.3%)	241 (25.0%)	104 (24.2%)
350 to 499	385 (13.3%)	29 (12.2%)	56 (7.8%)	67 (12.5%)	158 (16.4%)	75 (17.4%)
500+	439 (15.2%)	16 (6.8%)	47 (6.5%)	61 (11.4%)	201 (20.9%)	114 (26.5%)
Missing	2 (0.1%)	0 (0.0%)	0 (0.0%)	0 (0.0%)	1 (0.1%)	1 (0.2%)
Median CD4 nadir, cells/mm^3^ (IQR)	224 (107, 383)	168 (81, 287)	182 (97, 283)	216 (98, 341)	268 (120, 456)	319 (153, 507)
WHO clinical stage at diagnosis						
WHO stage 1	1080 (37.4%)	45 (19.0%)	206 (28.5%)	234 (43.7%)	362 (37.6%)	233 (54.2%)
WHO stage 2	899 (31.1%)	71 (30.0%)	220 (30.5%)	162 (30.3%)	332 (34.4%)	114 (26.5%)
WHO stage 3	632 (21.9%)	81 (34.2%)	218 (30.2%)	98 (18.3%)	172 (17.8%)	63 (14.7%)
WHO stage 4	110 (3.8%)	13 (5.5%)	44 (6.1%)	19 (3.6%)	26 (2.7%)	8 (1.9%)
Unknown/missing	167 (5.8%)	27 (11.4%)	34 (4.7%)	22 (4.1%)	72 (7.5%)	12 (2.8%)

### 3.1 CD4

**Figure 1:**
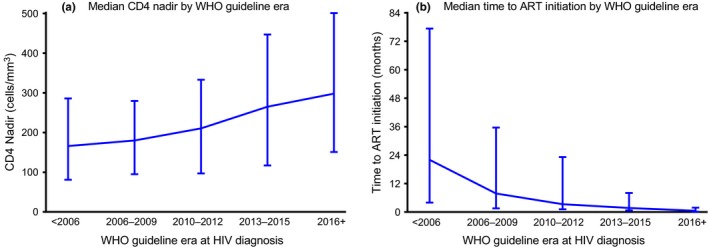
Medians and interquartile ranges for **(a)** CD4 nadir and **(b)** time from HIV diagnosis to ART initiation, by World Health Organization guideline. Bars represent interquartile range.

The median CD4 nadir in the study population increased from 166 (interquartile range (IQR): 81 to 286) cells/mm^3^ among participants diagnosed prior to 2006 to 298 (IQR: 151 to 501) cells/mm^3^ among those diagnosed ≥2016 (Figure 1a). Of the 237 participants diagnosed prior to 2006, 141 (59.5%) had CD4 nadir <200 cells/mm^3^. In contrast, among 429 participants with a recorded CD4 nadir diagnosed since 2016, 136 (31.6%) had CD4 nadir <200 cells/mm^3^ and 114 (26.5%) started ART with CD4 nadir ≥500 cells/mm^3^.

### 3.2 Time to ART initiation

**Figure 2:**
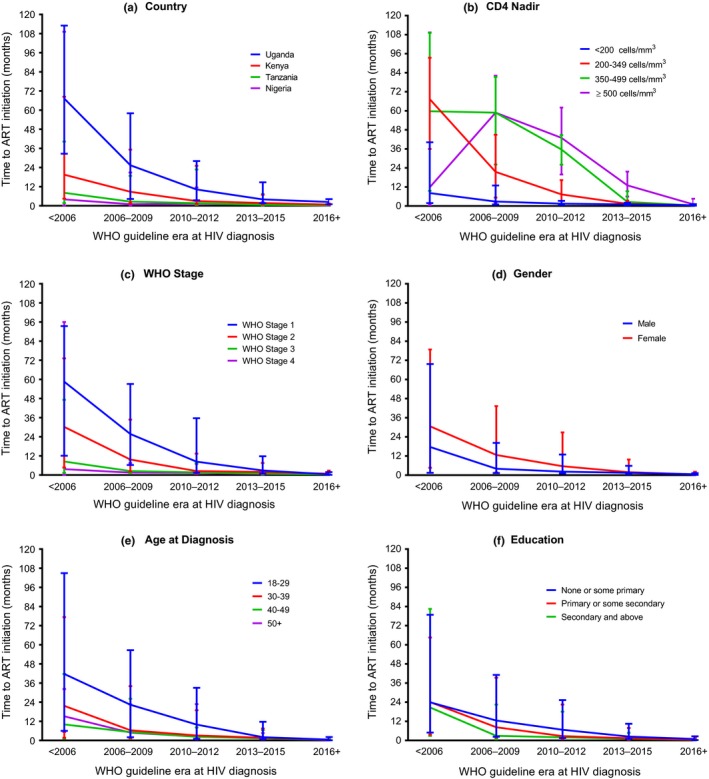
Median time to ART initiation by **(a)** country, **(b)** CD4 nadir, **(c)** World Health Organization clinical stage, **(d)** gender, **(e)** age, **(f)** education. Bars represent interquartile range. WHO, World Health Organization.

Time to ART initiation decreased as WHO guideline era at diagnosis increased (Figure 1b). Participants diagnosed prior to 2006 had a median time of 22.0 months (IQR: 4.0 to 77.3) between diagnosis and ART initiation. The median time to ART initiation decreased to 7.8 months (IQR: 1.5 to 35.6) for those diagnosed between 2006 and 2009. From 2016 on, the median time to ART initiation was less than a month after HIV diagnosis (median: 0.5 months; IQR: 0.2 to 1.8). Over the total duration of the study, participants receiving care from the Uganda site had the longest median time to ART initiation at 7.6 months (IQR: 2.3 to 26.4) while participants receiving care in Nigeria had the shortest median time to ART initiation at 0.9 months (IQR: 0.3 to 6.1). Among those diagnosed prior to 2006, median time to ART initiation ranged from a high of 67.3 months in Uganda (IQR: 32.7 to 113.0) to a low of 4.0 months in Nigeria (IQR: 0.2 to 109.2; Figure 2a). By 2016, time to ART initiation was under three months for all countries with Uganda having the longest median time to ART initiation at 2.4 months (IQR: 1.0 to 4.2).

Stratifying by CD4 nadir and era we found a decrease in time to ART initiation by WHO guideline era (Figure 2b). For each WHO guideline era, median time to ART initiation was shortest for those participants with CD4 nadir <200 cells/mm^3^ ranging from a median time of 8.2 months (IQR: 1.8 to 40.2) for those diagnosed before 2006 to 0.5 months (IQR: 0.2 to 1.2) for those diagnosed in 2016 and later. Stratifying by WHO stage and era we saw that participants at WHO stages 3 or 4 had the shortest ART initiation times across all WHO guideline eras (Figure 2c). Among participants with WHO stage 4, median time to ART initiation was 3.7 months for those diagnosed before 2006 (IQR: 0.1 to 96.0) and decreased to 0.6 months among those diagnosed in 2016 and later (IQR: 0.4 to 1.5). Stratifying by gender and era we found a decrease in time to ART initiation by WHO guideline era with females having a longer time to ART initiation compared to males among those diagnosed before 2006 (30.6 vs. 17.6 months). By 2016 females and males had a median time to ART initiation of 0.6 and 0.5 months respectively (Figure 2d). When stratifying by age and era there was a decrease in time to ART initiation by WHO guideline era and for each era, participants between 18 and 29 years had the longest median time to ART initiation (Figure 2e). Stratifying by education and era, participants with none or some primary education only had the longest median time to ART initiation across all WHO guideline eras (Figure 2f).

In the unadjusted models era, CD4 nadir, WHO stage, gender, country, age at diagnosis and education were all significantly associated with time to ART initiation (Table 2). Notably, compared to those diagnosed before 2006 all eras had increased rates of time to ART initiation with the largest HR in 2016 (2016 HR: 10.16; 95% CI: 8.38 12.33). The rate of ART initiation was highest for CD4 nadir <200 cells/mm^3^ with decreasing rates as CD4 nadir increased.

**Table 2:** Factors associated with time to ART initiation among people living with HIV enrolled in AFRICOS

	Unadjusted HR	Adjusted HR
	HR (95% CI)	HR (95% CI)
Era		
<2006	Ref	–
2006 to 2009	1.97 (1.67 to 2.32)	2.12 (1.79 to 2.50)
2010 to 2012	2.92 (2.45 to 3.49)	3.86 (3.22 to 4.63)
2013 to 2015	4.79 (4.03 to 5.70)	7.57 (6.32 to 9.07)
2016+	10.16 (8.38 to 12.33)	18.86 (15.39 to 23.10)
CD4 nadir		
<200	Ref	–
200 to 349	0.70 (0.63 to 0.76)	0.72 (0.66 to 0.80)
350 to 499	0.57 (0.50 to 0.64)	0.48 (0.42 to 0.55)
500+	0.51 (0.45 to 0.58)	0.32 (0.28 to 0.37)
WHO stage		
1	Ref	–
2	1.12 (1.02 to 1.23)	1.07 (0.97 to 1.18)
3	1.39 (1.26 to 1.55)	1.36 (1.22 to 1.52)
4	1.31 (1.07 to 1.62)	1.41 (1.14 to 1.74)
Gender		
Male	Ref	–
Female	0.81 (0.75 to 0.88)	
Country		
Uganda	Ref	–
Kenya	1.31 (1.18 to 1.46)	1.44 (1.29 to 1.60)
Tanzania	1.73 (1.52 to 1.96)	1.55 (1.36 to 1.77)
Nigeria	1.70 (1.45 to 1.99)	1.86 (1.58 to 2.19)
Age at diagnosis		
18 to 29	Ref	–
30 to 39	1.34 (1.21 to 1.47)	1.18 (1.07 to 1.30)
40 to 49	1.61 (1.44 to 1.80)	1.36 (1.22 to 1.52)
50+	1.70 (1.47 to 1.98)	1.38 (1.19 to 1.61)
Education		
None or some primary	Ref	–
Primary or some secondary	1.11 (1.01 to 1.21)	
Secondary +	1.13 (1.02 to 1.24)	

CI, confidence intervals; HR, hazard ratios; WHO, World Health Organization.

In the adjusted model, gender and education were no longer significantly associated with time to ART initiation. Participants diagnosed after 2006 had increased rates of ART initiation compared to those diagnosed before 2006 (Table 2). The rate of ART initiation remained the lowest for a CD4 nadir ≥500 cells/mm^3^ after adjustment for other factors (HR: 0.32; 95% CI: 0.28 to 0.37). Age at diagnosis also was significantly associated, with the youngest participants 18 to 29 years having the lowest rates of ART initiation compared to all other age groups.

In a sensitivity analysis restricted to participants with complete HIV diagnosis dates, we found similar results with a percent change <5% for all coefficients as compared to the main analysis that also included participants with only a known diagnosis year and or month and year. Rerunning the model using calendar year at diagnosis, there was an increased HR as year increased (HR ≥ 2016 vs. <2006: 15.0; 95% CI: 11.4 to 19.7). 

The interval between HIV diagnosis and ART initiation has decreased over time among PLHIV in this cohort and most are initiating ART within two weeks of diagnosis in the current era. Despite barriers such as healthcare capacity and societal norms, these data suggest that the programmatic shift to “test and treat” has been at least partially effective 13.

While progress has been made in all four African countries evaluated in these analyses, we saw significant differences by country in median time to ART initiation within each WHO guideline era. Country‐specific guidelines do not necessarily keep apace with evolving WHO guidelines and individual countries may also vary in their approach to HIV testing 14. One prior multi‐country cohort study described gender‐based regional variations in ART regimen selection, changes and discontinuations and found that median CD4 count was consistently lower in West Africa as compared to East Africa 15. In contrast, we found that the Nigerian site had the shortest time to ART initiation, perhaps reflecting that the Nigerian sites are the only urban sites in our cohort, which may allow for easier access to care. Additionally, the quality of care and intensity of support varies from programme to programme. Among the AFRICOS clinics, Nigeria has better‐funded referral facilities that are staffed by doctors as compared to Tanzania, which has a more basic facility with fewer financial resources that is staffed mostly by non‐physician providers.

CD4 nadir at ART initiation increased over the study period, in line with changes in WHO guidelines advising treatment at progressively higher CD4 levels. However, even in the current era, many individuals initiated treatment with low CD4, including about one‐third who initiated ART at an AIDS‐defining CD4 of <200 cells/mm^3^. Similar to our findings, a review of 17 ART programmes in 12 countries in Sub‐Saharan Africa found that while CD4 at ART initiation was increasing, for many participants it remained below recommended guidelines at the time 16. These findings highlight the need to diagnose HIV earlier while improving access to immediate ART after diagnosis. In our study, most participants in the current era started ART within weeks of HIV diagnosis, so particular attention must be paid to identifying barriers and gaps in diagnosing HIV earlier in the course of infection 17. Other studies have similarly found that diagnosing individuals with HIV before disease progression is a major challenge in the care cascade 18, 19.

We also found that time to ART initiation differed significantly by age. While time to ART initiation decreased over time among participants 18 to 29 years it was significantly longer than the other age categories for each WHO era. Other studies have similarly found that younger age groups are slower to initiate ART, highlighting the need for better linkage to care in younger adults 20, 21, 22. These findings also suggest that different strategies may be needed to link this key population to care.

Strengths of this study included the large sample size representing four countries in sub‐Saharan Africa. Diagnosis dates and, to a lesser extent, ART start dates were sometimes extracted from medical records rather than ascertained prospectively, resulting in missing data particularly in earlier WHO guideline eras. Additionally, for participants who initiated ART after the enrolment visit we only have a month and year for ART initiation limiting the precision of our time to ART estimate which is a particular limitation in the era of test and treat. Since most participants were diagnosed and started ART prior to study enrolment we could not assess for competing risks for ART initiation, such as loss to follow‐up and death. This also limited our ability to adjust for other important factors in the decision to initiate ART such as pregnancy, tuberculosis co‐infection and CD4 at HIV diagnosis or enrolment in care. In our population, enrolment years differed by site with more participants enrolled in the earlier years in Uganda versus later enrolment at the Nigerian sites, perhaps in part explaining the differences in time to ART initiation between these two countries. Study participants may also differ from the general population and our results may not be generalizable to populations receiving routine care in other centres. In addition to this selection bias, generalizability of the findings may also be impacted by survival bias particularly for those diagnosed before 2006 as they likely represent a subset with better survival than the general population of PLWH. For ease of analysis and interpretation, we evaluated temporal trends using WHO guideline eras rather than country specific changes in guidelines, which often lag variably behind updates to WHO guidelines, thereby explaining some of the country level differences in time to ART 14. However, a sensitivity analysis using calendar year as the key independent variable of interest yielded similar results.

## 4 Conclusions

In line with changes in WHO guidelines, time to ART initiation decreased substantially across four countries in sub‐Saharan Africa over the last decade. Despite progress with shortening the interval between HIV diagnosis and ART initiation, there are still participants starting ART with low CD4 counts in the current era, suggesting that improved screening and case‐finding efforts are needed to diagnose HIV earlier in the course of disease. Our findings also highlight the need to target young adults with different methods to increase early diagnosis and linkage to care within this demographic. Decreasing time to ART initiation is an important public health goal not only for individual health but also for prevention of secondary transmission to partners.

## Competing interest

The authors have no conflicts of interest to disclose.

## Authors' contributions

JO, JM, EB, FK and YA collected the research data. JA, CP and MR designed the research study. AE analysed the data. AE, TC and PC wrote the paper. All authors have read and approved the final manuscript.
